# The Role of *Staphylococcus aureus* YycFG in Gene Regulation, Biofilm Organization and Drug Resistance

**DOI:** 10.3390/antibiotics10121555

**Published:** 2021-12-19

**Authors:** Shizhou Wu, Junqi Zhang, Qi Peng, Yunjie Liu, Lei Lei, Hui Zhang

**Affiliations:** 1 Department of Orthopedics, West China Hospital, Sichuan University, Chengdu 610041, China; wushizhou1990@wchscu.cn (S.W.); zhangjunqi2@stu.scu.edu.cn (J.Z.); pengqi77@wchscu.cn (Q.P.); 2 West China School of Public Health, Sichuan University, Chengdu 610041, China; liuyunjie@scu.edu.cn; 3 West China Hospital of Stomatology, Sichuan University, Chengdu 610041, China

**Keywords:** biofilms, drug resistance, gene regulation, *Staphylococcus aureus*, YycFG pathway

## Abstract

Antibiotic resistance is a serious global health concern that may have significant social and financial consequences. Methicillin-resistant *Staphylococcus aureus* (MRSA) infection is responsible for substantial morbidity and leads to the death of 21.8% of infected patients annually. A lack of novel antibiotics has prompted the exploration of therapies targeting bacterial virulence mechanisms. The two-component signal transduction system (TCS) enables microbial cells to regulate gene expression and the subsequent metabolic processes that occur due to environmental changes. The YycFG TCS in *S. aureus* is essential for bacterial viability, the regulation of cell membrane metabolism, cell wall synthesis and biofilm formation. However, the role of YycFG-associated biofilm organization in *S. aureus* antimicrobial drug resistance and gene regulation has not been discussed in detail. We reviewed the main molecules involved in YycFG-associated cell wall biosynthesis, biofilm development and polysaccharide intercellular adhesin (PIA) accumulation. Two YycFG-associated regulatory mechanisms, accessory gene regulator (*agr*) and staphylococcal accessory regulator (SarA), were also discussed. We highlighted the importance of biofilm formation in the development of antimicrobial drug resistance in *S. aureus* infections. Data revealed that inhibition of the YycFG pathway reduced PIA production, biofilm formation and bacterial pathogenicity, which provides a potential target for the management of MRSA-induced infections.

## 1. Introduction

*Staphylococcus aureus* (*S. aureus*) is a life-threatening, opportunistic pathogen [1]. The social and financial burdens caused by *S. aureus*-related infections continue to increase globally [2]. *S. aureus* may infect host sites via implantable medical devices, including tubes and orthopedic or cardiac prostheses [3,4]. Evidence indicates that bacteria growing in biofilms better tolerate the action of antimicrobial drugs than planktonic cells, because biofilms facilitate cell–cell contact and concentrate nutrients, such as carbon and nitrogen [5,6]. Bacterial cells adjust their metabolism in response to environmental stress, such as exposure to antibiotics or extremes of temperature and pH. The two-component signal transduction system (TCS) enables microbial cells to regulate gene expression and the subsequent metabolic process associated with environmental changes [7].

YycFG, also designated as WalRK or VicRK, is a highly conserved TCS in Gram-positive bacteria with a low G-C content [8]. The YycFG system was first identified as part of a system that is essential for the survival of temperature-sensitive mutants of *Bacillus subtilis* [9] and *S. aureus* [10]. The YycFG TCS in *S. aureus* is essential for bacterial viability, the regulation of cell wall synthesis and physiological metabolic processes, but attempts to construct viable YycFG deletion mutants were not successful [11]. The histidine kinase YycG/WalK/VicK is anchored by a cytoplasmic membrane, and it monitors environmental stimuli. YycG responds to extracellular changes by transferring the phosphoryl group to activate the response regulator YycF/WalR/VicR, which results in modification of the expression of downstream target genes to adapt to environmental changes [12].

Biofilms are primarily protected by the extracellular matrix (ECM), which is composed of lipids, proteins, exocellular DNA (eDNA) and polysaccharides (EPS) [13]. Staphylococcal biofilm formation is mediated by polysaccharide intercellular adhesin (PIA), which is synthesized by the *ica* operon [14]. The *icaADBC* locus contains *icaA*, *icaD*, *icaB* and *icaC* genes, which are arranged into the operon [15]. Notably, enzymes that degrade PIAs were not found in *staphylococci* [16]. Recent data suggested a role for the *S. aureus* YycFG TCS in PIA matrix-associated drug resistance [17]. The *ica*-independent biofilms are more commonly observed in methicillin-sensitive *S. aureus* (MRSA) [18], whereas SarA-regulated PIA are more commonly observed in methicillin-resistant *S. aureus* (MSSA) biofilms. Previous studies investigated the *mecA* gene, which is responsible for methicillin resistance [19], but the potential mechanisms of the role of the *mecA* gene in PIA biosynthesis and biofilm formation in MRSA remain largely undetermined. Our previous data showed that YycF directly bound to the promoter regions of *icaA* genes and may regulate *icaA* expression, which suggests that biofilm polysaccharides and the subsequent antimicrobial drug resistance of *S. aureus* are targeted via the YycFG pathway [20]. We reviewed the bacterial factors involved in YycFG-dependent biofilm development, the impact of YycFG two-component systems in antibacterial agent resistance and the strategies for targeting *S. aureus* two-component systems in the management of this human pathogen.

## 2. Regulation of *S. aureus* YycFG Two-Component Systems

### 2.1. S. aureus Two-Component Systems

Fifteen TCSs in the whole genome of S. aureus are involved in the regulation of bacterial physiological metabolism [21,22]. The regulation of the S. aureus TCS inextricably affects bacterial antimicrobial resistance [23]. The TCS system is comprised of two components: (i) histidine protein kinase (HPK) receptor proteins, which are anchored to the cell membrane and sense external environmental stimuli, and (ii) response regulators (RRs), which regulate downstream target gene expression. After physical or chemical stimulation by the external environment, HPKs undergo phosphorylation. The phosphate group is transferred to the response regulator (RR). Phosphorylated response regulators directly bind to the promoter regions of downstream target genes and enhance the adaptive viability of bacteria [24,25].

### 2.2. Regulatory Roles of the YycFG TCS in Cell Wall Biosynthesis and Biofilm Formation

The yyc operon in S. aureus is comprised of four genes, yycF, yycG, yycH, yycI and yycJ. The membrane-associated regulator YycHI is an activator of YycG function in S. aureus [26]. Despite its essential role in bacterial viability, the physiological or mechanical signals sensed by YycG are not well understood. Recent structural analysis of the YycG PASCYT domain revealed a metal-binding site that binds zinc ions (Zn^2+^). The abrogation of metal binding increased YycG kinase activity and YycF phosphorylation, which indicates that Zn2+ binding negatively regulates YycFG [27]. The response regulator YycF participates in the regulation of cell wall synthesis and binds promoter regions that contain a conserved motif sequence [5′-TGT(A/T) A(A/T/C)-N5-TGT(A/T)A(A/T/C)-3′] of target genes via the helix-turn-helix domain of YycF [12,20].

The specific binding of YycF to promoter regions, including icaA, agr, sarA and sarX, modifies biofilms in an ica-dependent manner [8]. The ArlRS regulon is a global regulator of relevant genes, including cell wall-anchored adhesins, polysaccharide synthesis genes, cell wall remodeling genes, the urease operon and a large number of virulence factors [28,29]. The accessory gene regulator (agr) system greatly contributes to the formation of S. aureus biofilms [30], and the staphylococcal accessory regulator (SarA) drives biofilm organization by altering ica transcription and producing PIA [31,32]. The sarA gene in Staphylococcus epidermidis is an essential regulator of ica operon activation in biofilm formation [33]. The sarA gene in S. aureus is associated with bacterial oxidation sensing and virulence factors [34,35]. Notably, our recent study revealed that YycF directly regulated the predicted promoter regions of sarA, and YycFG TCS sensitized S. aureus biofilm formation to H_2_O_2_ exposure via the sarA pathway [20].

### 2.3. Regulatory Roles of the YycFG TCS in Response to Host Immunity

*S. aureus* is a major opportunistic human pathogen. S. aureus interacts with its human host as an innocuous member of the microbiota, or it breaks immune barriers to become an invasive pathogen [36]. Because YycFG TCS positively regulates certain virulence genes, including genes associated with host–matrix interactions (efb, emp, fnbA, and fnbB), cytolysis (hlgACB, hla, and hlb) and innate immune defense evasion (scn, chp, and sbi), its activity is closely linked to the host inflammatory response that is induced during infection [37]. However, virulence gene modulation is achieved via the coordination of another TCS, SaeSR (short for S. aureus exoprotein expression). YycF positively activates SaeSR TCS. SaeSR is a virulence factor regulation system that promotes lysis of polymorphonuclear leukocytes (PMNs) after phagocytosis, and it plays an essential role in S. aureus evasion of innate immunity [38]. During host cell–pathogen interactions, the innate and adaptive immune systems respond to S. aureus. Adaptive immunity amplifies the activity of innate immune cells and influences host susceptibility to S. aureus, and it is associated with chronic persistent infections [39]. S. aureus also developed evasion mechanisms from the adaptive immune response using virulence strategies. A second immunoglobulin binding protein (Sbi) is a cell wall-anchored surface protein that binds with the Fcγ portion of IgG, or it is secreted as a virulence factor that interferes with soluble complement factor C3, which manipulates adaptive immune responses to S. aureus [40]. Aurélia et al. [41] found that YycFG triggered cell wall turnover and degradation. Degradation of the cell wall via the NF-κB system resulted in the clearance of bacterial cells by the host immune system. Activated YycF stimulated the SaeSR TCS to increase the virulence gene expression involved in human–pathogen interactions and innate immune system evasion. Therefore, the fine tuning of YycFG plays an important role in determining the conditions of *S. aureus* infection.

## 3. The Impact of YycFG Two-Component Systems on Antibiotic Resistance

### 3.1. S. aureus Biofilms and Antimicrobial Drug Resistance

After biofilm formation due to intercellular aggregation, bacterial cell detachment caused by the action of bacterial products is critical for subsequent bacterial dissemination [42]. Staphylococcus biofilms are mediated by polysaccharide intercellular adhesin (PIA), which is synthesized by the *ica* operon [14]. PIA contributes to the facilitation of initial biofilm adherence [43]. PIA is a major component of the extracellular matrix that fixes staphylococcal cells within the biofilm mass, which increases resistance to mechanical force [44]. The classical and predominant adaptive modules from the TCS systems [45] modulate mechanisms associated with antibiotic resistance in most bacteria, including increased drug efflux, upregulation of antibiotic-degrading enzymes, biofilm production and enhanced cell permeability, which depend on the expression of corresponding downstream effectors [25]. Therefore, an understanding of the YycFG two-component system contributed to developments in our ability to combat *S. aureus* infections.

An animal study using co-infection models demonstrated that *ica*-positive *S. aureus* showed better in vivo survival than their corresponding *ica* mutants in wild-type mice [46]. The significant contribution of the *ica* genes toward *S. epidermidis* infection was confirmed using a *C. elegans* infection model, which indicated that *ica* genes were required for a lethal infection [47]. PIA, produced by *S. aureus* in vivo, significantly affected *S. aureus* systemic infections in mice [48]. *S. aureus* could grow synergistically with *Candida albicans* within biofilms [49]. These studies suggest that PIA production is important for infection and/or co-infection in vivo, especially *S. aureus*. Biofilm organization decreases the susceptibility to antimicrobial agents and/or antibiotics [25]. The potential mechanisms include persister cell formation, altered metabolic conditions and a decreased penetration into the biofilm extracellular matrix [50]. *S. aureus* persister cells were first observed in 1942 and demonstrated that non-growing dormant cells were resistant to penicillin [51]. Biofilms exhibit characteristics similar to persisted cells, and the biofilm matrix contributes to persistent infection by protecting bacterial cells from the immune system and antibiotics [52,53]. PIA, which accounts for most of the extracellular matrix, affects susceptibility to antibiotics by impairing penetration through the biofilm matrix. PIA likely enhances the horizontal transfer of drug resistance genes via its effect on cell-to-cell contact in biofilms [50] because *S. aureus* within biofilms was 1000 times more resistant than the bacteria in a planktonic state. Previous studies showed that *ica*-positive *S. aureus* strains had increased resistance to a variety of antibiotics, such as oxacillin, gentamicin, ciprofloxacin, levofloxacin, erythromycin and vancomycin, compared to *ica*-negative strains [54,55].

### 3.2. YycFG TCS-Associated Cell Membrane and Cell Wall Biogenesis Involvement in Drug Resistance

Antibiotic-resistant strains, particularly MRSA, are increasing in prevalence in hospital- and community-acquired infections and pose a significant threat to public health [56,57]. MRSA infections are responsible for substantial morbidity and lead to the death of 21.8% of infected patients annually [58]. To understand the mechanisms of resistance in MRSA strains, recent genomic studies demonstrated that antibiotic resistance in MRSA was primarily due to extensive modification of bacterial cell wall biogenesis [59,60]. These studies used a clinical MRSA strain to demonstrate that exposure to certain antibiotic combinations was associated with the development of mutations in specific genes, including *yycFG* [61]. Wu et al. isolated clinical MRSA strains from chronic osteomyelitis tissues. These MRSA strains demonstrated an accelerated growth rate compared to the MSSA strains and an accumulation of PIA matrix in the biofilms with the increased expression of the *yycF/G/H* and *icaA/D* genes [17]. The YycFG system is essential for *S. aureus* viability. Therefore, a recombinant plasmid shuttle vector was used to overexpress an antisense RNA and inhibit target gene expression, which led to the construction of antisense *yycG* RNA (AS*yycG*)-overexpressing MRSA strains. The AS*yycG* strains showed a reduction in biofilm formation and an increased antibiotic sensitivity to cefoxitin compared to MRSA strains, which may be attributed to altered PIA production [62]. To further investigate the regulatory roles of AS*yycG* in the pathogenicity of MRSA strains in vivo, a rat model of tibial osteomyelitis was developed and infected with MRSA- or AS*yycG*-overexpressing strains. The AS*yycG* strains exhibited suppressed invasive ability and pathogenicity in vivo*,* and the production of pro-inflammatory cytokines was reduced compared to MRSA strains [63].

To explain the potential mechanisms of AS*yycG* in regulating *S. aureus* biofilms, transcriptome analyses showed that AS*yycG* overexpression influenced the pathways associated with biofilm metabolism, virulence and glycolysis/gluconeogenesis utilization in *S. aureus*, including the *sarA* and *icaA* genes [20]. For the potential role of the response regulator YycF in biofilm formation and pathogenicity, endogenous antisense *yycF* RNA (AS*yycF*) was detected using a 5′ RACE assay. The over-production of AS*yycF* reduced YycF production and biofilm formation. Antibiotic sensitivity to vancomycin was significantly improved in AS*yycF*-overexpressing strains compared to MRSA strains. AS*yycF*-overexpressing MRSA strains exhibited suppressed invasion in a rat tibial infection model [63], which supports AS*yycF* as a supplementary strategy for the management of *S. aureus* and MRSA infections. Taken together, these data indicate that inhibition of the YycFG pathway reduced PIA production, biofilm formation and bacterial pathogenicity, which provides a potential target for the management of MRSA infections. The YycFG TCS was identified in the process of cell wall biosynthesis. Cell wall thickening and an aberrant division of septa are closely associated with YycFG [64]. LytM and SsaA play crucial roles in cell wall peptidoglycan crosslinking relaxation during the cell division process and are regulated by the YycFG system, which is required for cell viability. A previous study revealed lipid II as an essential component of the cell wall and a signal that is sensed by YycG kinase. Antibiotics, such as β-lactams, which characteristically target lipid II, activate YycFG TCS [65]. However, the efficacies of last resort agents, such as vancomycin, linezolid and daptomycin, in the treatment of serious MRSA infections are controversial.

Vancomycin is a glycopeptide antibiotic that inhibits cell wall synthesis via binding to the D-alanyl-D-alanine residue on the bacterial cell wall [66]. Ten types of vancomycin-intermediate resistance in *S. aureus* (VISA) were analyzed, and three types of heterogeneous vancomycin-intermediate *S.* *aureus* (hVISA) strains were sequenced using high-throughput techniques. Site mutations in the *yycFG* gene were detected in eight types of VISA and two types of hVISA strains, which exhibited the highest mutation frequency [67]. These results suggest that the *yycFG* gene plays an important role in the generation of VISA and hVISA strains. Jansen et al. reported that the insertion of an enhanced promoter sequence in the promoter region of the *yycFG* gene of the *S. aureus* VISA strain increased the expression of its downstream target genes and up-regulated bacterial cell wall biosynthesis, which increased antimicrobial drug resistance [68]. Domains of the *yycFG* gene of the VISA strain are affected by the mutation of a single nucleotide, such as the A96T mutation in the *yycF* gene, which is a mutation from base G to base A at site 24673 of the *S. aureus* genome. This mutated base is located in a conserved region of the *yycF* gene, and it is associated with a conformational change to protein phosphorylation regulation. These mutations decrease the activity of the YycFG protein and down-regulate the expression of bacterial autolytic enzymes, which inhibits the bacterial autolytic process [67]. The *yycHI* genes are downstream of *yycFG* and bind to the YycG histidine kinase receptor to interact with the YycFG pathway [26]. Auxiliary YycH and YycI are ‘connector’ proteins that physically interact with the YycG sensor kinase to form a ternary protein complex that activates the YycFG TCS. Mutation of these auxiliary proteins disrupted the integration of YycFG two-component networks and reduced vancomycin susceptibility in clinical VISA strains. The mutation rate of the *yycHI* gene in these strains was significantly higher than the vancomycin-sensitive *S. aureus* strain. Mutation of *yycHI* may lead to the enhancement of cell wall synthesis and enhance antimicrobial resistance [67], but further investigation is required.

Membrane-bound receptors and cognate cytosolic response regulators, such as the YycG receptor and the YycF regulator, are closely associated with a phospho-relay mechanism on initiation. Upon phosphorylated, the YycF response regulator plays a role in transcription factor binding to DNA and modulates associated gene expression to orchestrate several physiological functions involved virulence, cell wall metabolism and biofilm formation [37,69]. Daptomycin (Dap) is a cell membrane-targeting lipopeptide antibiotic that exhibits excellent antibacterial activities against susceptible Gram-positive pathogens [70]. The combination of Dap with calcium significantly reduced cell viability via cell membrane depolarization and permeabilization [71]. Because the YycFG TCS regulates the cell envelope and lipid metabolism-associated genes, including *atl*, *lytM*, *sceD*, *isaA*, and *ssaA*, it plays a fundamental role in cell membrane metabolism [8,65]. Because *S. aureus* extracellular genomic DNA (eDNA) is released from bacteria via cell lysis, the role of *S. aureus* autolysin, Atl, may be implicated in biofilm development, especially in initial attachment [72]. YycFG TCS maintains cell membrane fatty acid homeostasis [73], confers resistance to depolarization and/or permeabilization and contributes to daptomycin resistance (DAP-R). The development of DAP-R in *S. aureus* was observed clinically during therapy, and it is often associated with treatment failure [74]. DAP-R strains acquire a progressive accumulation of single nucleotide polymorphisms in the YycFG TCS of the *yycFGHI* operon, which is involved in key cell membrane events, and the *yyc* operon is involved in the generalized response to antimicrobials [71]. Clinical MRSA strains that emerged with daptomycin non-susceptibility were isolated to examine the influence of certain antibiotic combinations, including daptomycin with or without adjunctive clarithromycin, linezolid, or oxacillin, on the development of mutations in specific genes, including the multi-peptide resistance factor gene (*mprF*) and *yycFG*. Daptomycin alone or combined with other antibiotics resulted in mutations in *mprF* and *yycFG*, which suggests that combining daptomycin with different antimicrobials affects the mutational space required for daptomycin nonsusceptibility development [61]. These results indicate that the use of adjunctive antibiotic therapy in a clinical setting alters the mutational space permitted for drug resistance development, which warrants the exploration of novel targeted molecular treatments.

## 4. Targeting the *S. aureus* Two-Component Systems

### 4.1. Molecular Targets

Two-component systems integrate with other signaling molecules in bacteria via cross-activation with other transcription factors [75]. The staphylococcal accessory regulator (SarA) is a global regulator that controls the transcription of a wide range of virulence genes [76]. A novel inhibitor of SarA was designed to prevent *S. aureus* biofilm formation, and it was developed as a potential antimicrobial strategy in prosthetic joint infections [77]. Global SarA expression is linked to the YycFG TCS, which binds to the promotor region of the *sarA* gene [20]. This activation affects biofilm formation and the pathogenic and antimicrobial resistance potential of *S. aureus*, which offers a supplementary therapeutic target.

### 4.2. Non-Coding RNA Regulation

Two-component systems are integrated with other signaling molecules in bacteria, such as regulatory RNAs. RNA III is a “small” regulatory RNA (sRNA) in *S. aureus*, which is controlled by the AgrAC TCS, and participates in quorum sensing and pathogenicity “antisense regulation” via *spa*, *rot* or *hla* at the post-transcriptional level [78]. The formation of double-stranded RNA structures via base pairing of the 5’ or 3′ terminal sequences of target mRNAs with corresponding antisense RNAs provides positive translation initiation and mRNA stability and stimulates RNA degradation by RNase for transcription interference and attenuation [79,80]. Endonuclease RNase III plays a critical role in the latter effect via the cleavage of double-stranded RNA structures [81]. Our previous study showed that promoter regions of the RNase III–encoding gene (*rnc*) bound and were directly regulated by the YycF ortholog gene VicR in *S. mutans* [82]. Antisense regulation mechanisms were used to inhibit antibiotic resistance in bacterial infections using antisense oligonucleotides [83]. Notably, an endogenous antisense RNA base paired with *yycF* mRNA was identified in *S. aureus* [84], which belongs to a trans-encoded sRNA [79]. The length of the AS*yycF* operon is approximately 400 bp, and because endogenous AS*yycF* significantly downregulated the expression of YycFG TCS, it may restrict biofilm formation and reduce antibiotic resistance and pathogenesis. Therefore, this pathway should be considered as a supplementary strategy for the management of *S. aureus* and MRSA infections.

## 5. Concluding Remarks

**Figure 1:**
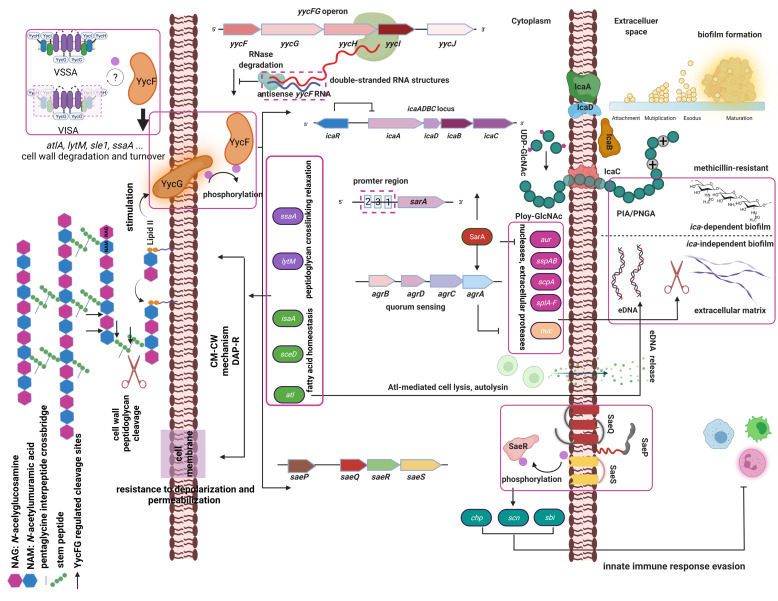
The only essential YycFG two-component regulatory system (TCS) is a large operon that comprises *yycFGHIJ* and influence antibiotic resistance in *S. aureus*. YycG is a sensor histidine-kinase compromised by two transmembrane sequences and a periplasmatic loop [37,85]. To sense and respond to specific environmental cues, YycG can auto-phosphorylate and transfer phosphoryl group to its cognate response regulator YycF inducing activities of biofilm formation, susceptibility to antibiotics such as vancomycin and daptomycin and innate immune system evasion. The *icaADBC*-encoded polysaccharide intercellular adhesin (PIA) or polymeric N-acetyl-glucosamine (PNAG) from UDP-N-acetylglucosamine (UDP-GlcNAc) contributes to *ica*-dependent biofilm development [16]. Besides *ica*-dependent biofilm, *ica*-independent extracellular matrix significantly contributes biofilm formation [86]. eDNA released by the major autolysin of *S. aureus* Atl in the lysis of bacteria and leads to enhanced biofilm formation [87]. The major global regulators, staphylococcal accessory regulator (*sarA*) is driven by three different promoters (P1, P2 and P3) [88]. SarA is a positive regulator of *agr* (Accessory gene regulator) quorum-sensing system including four genes, *agrBDCA*. Under the YycFG TCS control, SarA results in downregulation of proteases and the thermostable nuclease such as *aur*, *sspAB* (Staphylococcal serine proteases), *scpA* (Staphylococcal cysteine protease operon), *splA-F* (Serine protease-like proteins) and *nuc* (Thermostable nucleases), allowing for biofilm maturation [76,88]. This biofilm formation causes a relationship with methicillin resistance status [89]. Auxiliary proteins YycH and YycI play a positive role with YycG for a ternary protein complex to activate YycF activity triggering an increased gene expression of *atlA* (Autolysin), *sle1*(N-acetylmuramyl-L-alanine amidase), *lytM* (Lysostaphin-type peptidase), *ssaA* (Staphylococcal secretory antigen A), which contributes to cell wall (CW) metabolism and associated with clinical vancomycin-intermediate *S. aureus* (VISA) [26]. Peptidoglycan is an essential component for the bacterial cell wall. It is assembled from Lipid II. By “sensing” different levels of lipid II, YycFG TCS plays a fundamental role in peptidoglycan crosslinking relaxation associated genes, including *lytM*, *ssaA*. Cleavage sites for YycFG regulated cell wall hydrolases are indicated (black arrow) [64,71]. In addition, YycFG has been shown to regulate cell membrane (CM) lipid metabolism including *atl*, *sceD* (*Staphylococcus epidermidis* D protein), *isaA* (Immunodominant staphylococcal antigen A) to alter CM dynamics [71]. Both CW and CM mechanisms contribute to the development of daptomycin-resistance (DAP-R). SaeP and SaeQ are two auxiliary proteins from the *sae* (*S. aureus* exoprotein) operon involving in phosphatase activity of histidine sensor kinase SaeS and activated SaeS phosphorylates its cognate response regulator SaeR [90]. YycFG can trigger a response on SaeRS leads to higher virulence genes expression of *chp* (Chemotaxis-inhibiting protein), *scn* (Staphylococcal complement inhibitor) and *sbi* (Second binding protein of immunoglobulin), involving innate immune system evasion [41]. Therefore, YycFG play an important role in the state of commensal *S. aureus* as a pathogen. Trans-encoded sRNAs antisense *yycF* base-paired with *yycF* mRNA constructs as a double-stranded RNA structure and interferes YycFG TCS at the post-transcriptional level [79,84]. Created with BioRender.com.

YycFG TCS is the only essential TCS for *S. aureus* to adapt to a wide variety of environments. Many other resistance mechanisms, including cell wall peptidoglycan metabolism, cell membrane lipid metabolism and innate immune system evasion, are directly or indirectly regulated by YycFG (Figure 1). *S. aureus* acquired a collection of virulence factors that enabled the bacterial cells to colonize biotic and abiotic surfaces and form a biofilm. This biofilm structure allows *S. aureus* to sense and resist harsh environmental conditions, physical and chemical stimuli and antimicrobial drugs, thereby enabling S. aureus to contribute to chronic and recalcitrant infections. The *S. aureus* YycFG TCS and several global gene regulators coordinate important functions during the establishment and maturation of the biofilm. Despite recent advances in this field, the available data are generally limited to in vitro studies using laboratory strains. Most of the studies on *S. aureus* biofilms were performed under static conditions and do not account for environmental signals that may variably affect biofilms. The development of new models that mimic the processes during biofilm growth in human infections is critical for the study of the mechanisms that drive *S. aureus* biofilm production. Future novel and effective anti-infection therapies will likely include antimicrobial agents that exhibit antibiofilm properties. Exploration of the molecular targets present in *S. aureus* two-component systems and their gene regulators, such as the ica operon, sarA, atl, lytM, sceD, isaA, and ssaA, is worthwhile. Non-coding RNAs elucidated the post-transcription regulation of biofilm growth and bacterial resistance to antimicrobials. The designing of a nucleotide delivery system with high transfection efficiencies, favorable biocompatibility and safety are needed to target RNA interference and as a novel strategy to treat infections and tackle drug resistance.
